# Electrophysiological and Behavioral Responses to Overinclusion Following Preexposure to Social Threat

**DOI:** 10.1111/psyp.70107

**Published:** 2025-07-05

**Authors:** Xu Fang, Rudolf Kerschreiter, Yu‐Fang Yang, Michael Niedeggen

**Affiliations:** ^1^ Institute of Psychology and Behavior Henan University Kaifeng China; ^2^ Division of Experimental Psychology and Neuropsychology, Department of Education and Psychology Freie Universität Berlin Berlin Germany; ^3^ Division of Social, Organizational, and Economic Psychology, Department of Education and Psychology Freie Universität Berlin Berlin Germany

**Keywords:** cyberball, expectancy violation, loss of control, overinclusion, preexposure, sociometer theory

## Abstract

Previous research demonstrated that experiencing a social threat can affect how individuals process subsequent social threats. This “preexposure effect” suggests that different social threats, such as loss of control and exclusion, interact within a common cognitive system. In this study, we extended the preexposure effect to examine how a prior social threat influences subsequent positive social interactions. Specifically, we investigated how the experience of a loss of control affects neural processing and retrospective evaluations of subsequent overinclusion. Our findings revealed that the event‐related brain potentials (ERPs) previously related to the processing of exclusion and overinclusion (P3 effect) remained unaffected by the preexposure threat. However, the preexposure threat influenced the expression of frontal positivity (P2) which has been previously associated with the processing of social rewards. In addition, we observed that the expression of the perceived threat to belonging and negative mood depends on the continuation—or discontinuation—of the preexposure threat in the subsequent period of overinclusion. These results question the idea of a continuum of social participation ranging from exclusion to overinclusion. The latter appears to be more closely linked to the perceived valence of cues related to social inclusion.

## Abstract 2

This study reveals that social threats, like loss of control, affect the processing of following positive social interaction, here overinclusion. ERP data indicate that a preexposure to a threat affects the valence of inclusionary signals, and therefore reduces their ameliorative social function.

## 1 Introduction

The need to belong represents an inherent human imperative that drives individuals to seek acceptance and avoid rejection (Baumeister and Leary 1995; DeWall et al. 2011). Belonging to a group not only offers a sense of security but also provides access to resources that are vital for survival (Allen et al. 2021; Lancaster 1986). The concept of belonging can be delineated along a continuum (Williams et al. 2000), similar to a “sociometer” (Cameron and Stinson 2017; Leary et al. 1995), ranging from threatened belonging to fair to enhanced belonging. Threatened belonging is typically caused by the exclusionary behavior of individuals or groups (Williams 2009). Overinclusion, in contrast, is defined as an enhancement in social interaction with others (Williams et al. 2000). It could be considered at the opposite end of the sociometer scale. Accordingly, the need to belong is satisfied to an even larger degree (Hay et al. 2023).

**FIGURE 1:**
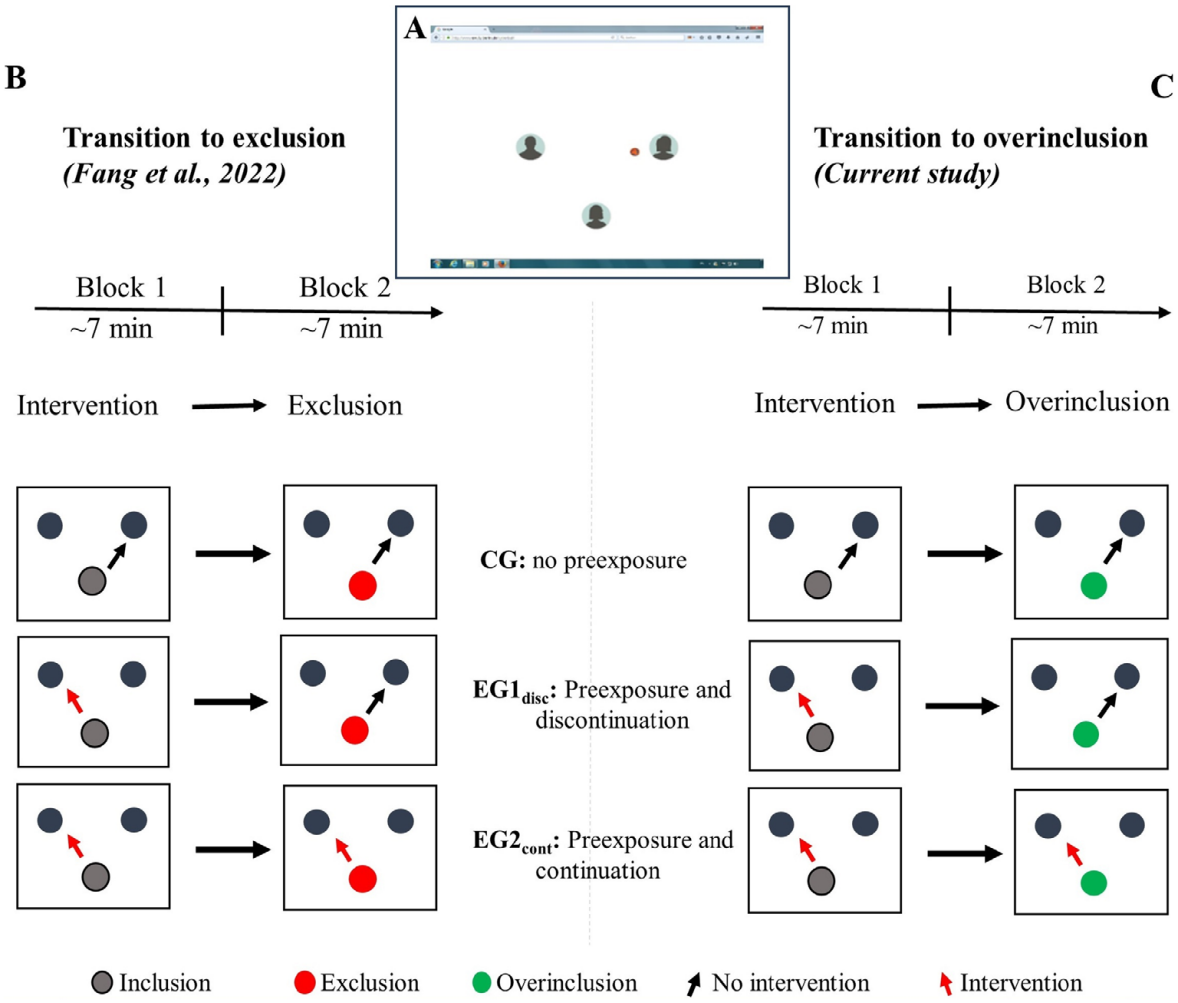
(A) Setup: On the computer screen, three avatars (sketches of human heads) represented one participant (middle position) and two putative co‐players. The symbol of the ball in spatial proximity to one avatar signaled the possession of the ball. (B, C) Design: Each participant ran through two experimental blocks of the Cyberball game. In block 1, all participants were included fairly, but participants assigned to experimental groups experienced partial loss of control (indicated by the red arrow). In block 2, participants in all groups received the ball less frequently (indicated by the red dot) in the previous study (Fang et al. 2022) or more frequently (indicated by the green dot) in the current study, and loss of control continued in the second experimental group (EG2_cont_).

To systematically assess the effects of exclusion and overinclusion on behavioral and physiological responses, it is advantageous to use a unified experimental paradigm. A promising candidate is the Cyberball paradigm (Williams and Jarvis 2006), a virtual ball‐tossing game (see Figure 1A). In this game, participants are led to believe that they are engaging in an online game with two co‐players, whereas, in reality, these “co‐players” are computer‐generated. By manipulating the probability of participants' ball reception, different levels of social involvement can be induced, from exclusion to overinclusion.

The subjectively perceived level of social participation is typically measured using the Need‐Threat Questionnaire (NTQ, Williams 2009), which has been used to validate four basic need threats (belonging, control, self‐esteem, and meaningful existence) and negative mood. When ball reception falls below fair standards, participants report stable increased social need threats and negative mood (Hartgerink et al. 2015). In contrast, increasing ball reception beyond fair parameters yields opposite effects: basic social needs are fulfilled (Niedeggen et al. 2014; Venturini et al. 2016) and individuals' mood is affected (De Waal‐Andrews and Van Beest 2020; Kawamoto et al. 2012; Niedeggen et al. 2014; Venturini et al. 2016). These effects have been confirmed in a recent meta‐analytic review (Hay et al. 2023). The results of these self‐reports appear to be in agreement with the sociometer model (Mahadevan et al. 2020). According to this model, reduced ball reception affects self‐esteem and consequently threatens the need for belonging (Leary and Baumeister 2000). In contrast, higher self‐esteem, here induced by overinclusion, could prompt a confirmation of the subjective sense of social inclusion.

Whereas questionnaire methods are based on retrospective reports, electrophysiological methods, such as event‐related brain potentials (ERPs), allow us to monitor processes immediately related to the processing of exclusion and overinclusion. In most studies, the event “ball reception” of the player serves as a probe for the functional state of the participants (for review: see Vanhollebeke et al. 2023). In an exclusionary Cyberball setup, a reduction in ball receptions is often characterized by an increase in P3 amplitude (Jenkins and Obhi 2020; Niedeggen et al. 2023; Themanson et al. 2013). It is crucial to note that the increased P3 amplitude is not a mere reflection of a change in probability, but is related to a violation of the expected participation (Mills et al. 2023; Weschke and Niedeggen 2015). This notion becomes evident when a reduction in involvement in the Cyberball game can be anticipated by increasing the number of co‐players (Weschke and Niedeggen 2015). In this case, a reduction of ball reception did neither evoke threats to social needs nor did the P3 amplitude increase.

As for the ERP effects of overinclusion, previous studies (Niedeggen et al. 2014; Weinbrecht et al. 2018) showed a decrease in P3 amplitudes as the frequency of ball receptions increases. The direction of this P3 effect is in line with the sociometer theory (Leary et al. 1995) and might be linked to an internal gauge monitoring the extent of social acceptance. Meanwhile, the transition to overinclusion also elicited an additional component not observed in exclusionary settings—a fronto‐central positivity (P2 component). This component, not prominent in an exclusionary Cyberball, may signal the processing of social reward (Cao et al. 2015; Weinbrecht et al. 2021).

Besides the sociometer theory (Leary and Baumeister 2000; Leary et al. 1995), the current study is based on the idea that social threats are processed in a common cognitive system (Fang et al. 2024, 2022; Niedeggen et al. 2023; Proulx et al. 2012). More specifically, the idea implies that the deviation from subjective expectations is not only a prerequisite for a threat to the need for belonging (Sommer and Rubin 2013) but does extend to other social needs. Empirical evidence has been provided using a modified version of the Cyberball paradigm (Niedeggen et al. 2019): Here, a loss of control (where ball throws were casually overruled by a putative “supervisor”) is considered an aversive event that violates subjective expectations for “control”. In line with this idea, the loss of control triggers P3 effects corresponding to those triggered by social exclusion. These findings align with the inconsistency compensation approach (Proulx et al. 2012), suggesting that a common expectancy system is activated by the violation of various subjective beliefs, goals, and expectations.

The common basis of the processing of different social threats implies that the experience of one social threat might affect the processing of a subsequent different social threat. This has recently been demonstrated by Niedeggen et al. (2023) who reported interaction effects induced by the simultaneous processing of threats to control and belonging. In addition, Fang et al. (2022) demonstrated that this is also the case when one social threat is processed clearly before a subsequent different social threat. To test the effects of subsequent social threats, Fang et al. (2022) established a new experimental setup (“preexposure setup”): Here, participants played two blocks of Cyberball (see Figure 1B). In the control group, fair treatment in the first block was followed by exclusion in the second block. In two experimental groups, participants encountered a specific social threat, loss of control, in the first block—which defines the preexposure. In the second block, these participants experienced exclusion. In the control group, this transition elicited a clear P3 effect (defined by the amplitude difference between block 1 and block 2). However, this effect was modulated by preexposure: In contrast to the control group, P3 effects were significantly reduced in the two experimental groups with preexposure. Notably, the expression of the decreased P3 effect in the experimental groups depended on the fate of the preexposure threat: The P3 effect was less expressed if the preexposure threat was discontinued (labeled as EG1_disc_) in contrast to a continuation of the first threat in block 2 (labeled as EG2_cont_). This pattern of results is depicted in Figure 3B (left pane).

The ERP data (Fang et al. 2022) indicated that preexposure biases the expectation of upcoming social interaction: The onset of exclusion was less surprising in the experimental group (EG2_cont_) compared to the control group (CG), and due to the continuation of the threat in the second block, no adjustment of the expected interaction was necessary. In contrast, the offset of the preexposure threat in the other experimental group (EG1_disc_) may provide a pleasurable experience (Willems and Vervliet 2021) which is incongruent with the biased expectation. Therefore, adjustment processes—reflected in the P3 amplitude—are reinstated.

Notably, the self‐reports (Fang et al. 2022) were primarily determined by the offset of the preexposure threat: If the first threat overlapped with the second threat in the second block (EG2_cont_), participants reported similar levels of threat to belonging and negative mood as the control group that had not experienced any threat in the first block. In contrast, if the first threat was discontinued (i.e., offset of the preexposure) this significantly reduced the threat to belonging and negative mood reported after the second block. In other words, the retrospective self‐reports were especially sensitive to the offset of the first threat after block 1.

The current study also employs the preexposure approach to investigate how the processing of social overinclusion is influenced by prior experiences of loss of control within the Cyberball paradigm. As mentioned above, preexposure to a social threat is assumed to bias individuals' expectations for the upcoming social interaction, and this bias modulates the expression of the P3 effect (Fang et al. 2022, 2024). If exclusion and overinclusion define the endpoints on a continuum, then a “priming” process that applies to exclusion should also apply to overinclusion. Accordingly, we reasoned that experiencing a loss of control can affect the processing of an upcoming overinclusion (Fang et al. 2022). Based on the previous studies, we stated the following two hypotheses for the ERP effects and the self‐reports:

The first hypothesis is focused on the P3 effect: The transition to overinclusion will reduce the P3 amplitude. This P3 effect will depend on the previous experience of a social threat, and on its continuation: In the control group, the expected social participation is not biased, and the P3 effect is clearly expressed. In the experimental groups, preexposure to a social threat will increase the expression of the P3 effect. However, if the transition to overinclusion coincides with the offset of the threat (discontinuation), the P3 effect will be further increased because the bias in expected social involvement is further widened.

Following previous self‐report findings (Fang et al. 2022), we expect that the response pattern will be primarily determined by the offset of the preexposure threat, but not a bias in the expected social involvement. Correspondingly, we reasoned for the second hypothesis that the decrease of the threat to belonging and negative mood is not primarily due to the onset of overinclusion (Hay et al. 2023), but to the discontinuation of the preexposure threat which serves as a pleasurable experience. Consequently, effects will be mostly marked in the experimental group.

Besides the P3 effect, another exploratory analysis will focus on the P2 component. Two previous studies (Niedeggen et al. 2014; Weinbrecht et al. 2021) reported congruently that the amplitude of the P2 was significantly increased by a transition from inclusion to overinclusion. According to the authors, this effect might indicate that overinclusion serves as a social reward. Although the current study primarily focused on expectancy‐based processes related to the P3, we also tested the reliability of the P2 effect and its relation to the preexposure threat. As previous results did not allow a prediction on the effect of a preexposure threat, no directional hypotheses have been stated.

## 2 Methods

The research protocol received approval from the local ethics committee of Freie Universität Berlin (No. 006.2019, 15 May 2019). Written informed consent was obtained from all participants before and after testing, in accordance with the Declaration of Helsinki. Preprocessed EEG data and the source code of the experimental procedure can be accessed at: https://osf.io/qpajf/ (accessed on August 20, 2024). All manipulations, measurements, and exclusion criteria are thoroughly reported.

### 2.1 Participants

Sample size was predetermined using G*power software (Erdfelder et al. 1996). The calculation was focused on a 3 × 2 interaction effect for the P3 amplitude of a between‐factor “group” (3 levels) and a within‐factor “block” (2 levels). To detect a medium effect (*f* = 0.20, adjusted to the taxonomy, Cohen 2013), at least 66 participants were required with a statistical power of 80%, using an *F*‐test with alpha at 0.05. A total of 72 participants were recruited from universities in the Berlin area. These participants were selected based on their normal or corrected‐to‐normal visual acuity, without concurrent acute psychological disorders or medical conditions that could potentially confound the study results. Mother tongue differed between participants (German *n* = 9, English *n* = 7, and Chinese *n* = 56). The language of instruction was adapted accordingly. All of them were randomly assigned to one of three groups: control group without preexposure (CG), experimental group 1 with discontinued first threat (EG1_disc_), and experimental group 2 with continued first threat (EG2_cont_), each consisting of 24 participants. Post hoc inspection confirmed that participants with different mother tongues were almost equally distributed across three groups (see Table S1). After rigorous correction of EEG artifacts (see below section: Data Analysis), three participants were excluded from the study. The final sample consisted of 69 participants (42 females, 27 males) with an age range of 19–39 years (*M*_age_ = 26.41 years, SD_age_ = 3.82 years). Each group comprised 23 participants (CG: 13 females; EG1_disc_: 15 females; EG2_cont_: 14 females).

### 2.2 Task and Design

The Cyberball experiment was implemented in PsychoPy2 (v1.85.6 (Peirce 2007)). The original setup (Williams and Jarvis 2006) was adapted to an EEG‐compatible Cyberball paradigm (Gutz et al. 2011; Niedeggen et al. 2019) that allowed the manipulation of probability for ball reception and control. The preexposure setup combining these two threats (exclusion and loss of control) has been established in previous experiments (Fang et al. 2022).

The objective of this study required experimental manipulation of the recipient of the individuals' ball throw (control) and the probability of ball reception (overinclusion). The setup of the Cyberball game, as illustrated in Figure 1A, featured three avatars (depicted as sketches of human heads) on the computer screen, representing players who were putatively connected via the Internet. These avatars were positioned at a spatial distance of 3° at a viewing distance of 120 cm. Participants' avatar was always centered horizontally in the lower section of the screen. The avatars representing two putative co‐players were centered vertically. To simulate a ball‐tossing game, an appropriate ball icon was presented. The appearance of the ball near an avatar signaled that the corresponding player possessed the ball. If participants had the ball, they were required to freely pass it to one of the other co‐players by pressing the corresponding arrow key (left or right) on the keyboard. No specific time limit was imposed for their response. Following the participants' action, the ball disappeared for 500 ms before reappearing beside one of the co‐players. This reappearance duration ranged from 400 to 1400 ms, to reinforce the belief that they were playing with humans.

All participants were informed that their primary task was the visual imagery of the ball‐tossing game and that the Cyberball game served as a tool for measuring their mental visualization skills. To support this cover story, participants were required to complete the Vividness of Visual Imagery Questionnaire (VVIQ, Marks 1973) before engaging in the ball‐tossing game. Data from this questionnaire were not considered in the analysis.

After completing the questionnaire and the attachment of electrodes, participants were instructed to position themselves in front of a computer monitor. Their chins were rested on a chin rest that could be adjusted for height. Participants were reminded to visualize the ball‐tossing scenario throughout the Cyberball game. Subsequently, participants were presented with six different avatars and asked to pick one representing the participant during the game. This freedom to pick an avatar is aimed to evoke heightened arousal during the game (Lim and Reeves 2009). The written instruction also informed the participants that an independent “supervisor” might intervene in their ball‐throwing decisions throughout the game. Such interventions would result in the ball reception of the non‐intended co‐player. It was clarified that the supervisor was not one of the co‐players engaged in the game. No further information on the supervisor was provided.

To facilitate a sense of familiarity and establish a baseline of expectations, participants started the game with a brief practice session lasting about 3 min. This practice session involved a total of 100 ball throws, with each player having a 33% probability of receiving the ball (inclusion condition), and no intervention occurred. Following the practice session, participants proceeded to engage in two experimental blocks, each comprising 200 ball throws. Each block lasted around 7 min, and a short break of 2 min was provided between blocks (see the design in Figure 1C). In three groups, social inclusion was consistently provided in block 1, comprising 200 ball throws with a 33% probability of each player receiving the ball, resulting in approximately 66 ball possessions per player. In contrast, social overinclusion was consistently provided in block 2, consisting of 200 ball throws with a 46% probability of participants' ball reception, leading to a total of 92 ball possessions by participants.

In the experimental groups (EG1_disc_ and EG2_cont_), preexposure to a threat was implemented: In both groups, the supervisor intervened during block 1 at a rate of 30%. In block 2, no interventions occurred in EG1_disc_, while interventions continued to occur in EG2_cont_ at the same probability as in block 1.

Immediately following block 2, participants were presented with a series of questionnaires. The corresponding questions were presented on the screen, and a rating was provided on a 7‐point Likert scale ranging from “−3” (indicating stronger feelings in block 1) to “3” (indicating stronger feelings in block 2). These questionnaires encompassed four types: (1) estimations of the frequency of ball reception or intervention; (2) the perceived threat to “belonging” and “control” (three items each, e.g., “I felt disconnected”, adapted from (Williams 2009; Williams et al. 2000)); (3) assessments of negative emotions (eight items, e.g., “I felt sad”, adapted from (Watson et al. 1988; Williams 2009)); and (4) ratings of the self‐assigned personal and social power (two items each, e.g., “I felt independent”, adapted from (Lammers et al. 2009)).

To accommodate participants with different native languages, the self‐report measures were administered in the respective languages of German, English, or Chinese. The German (Grzyb 2005; Weinbrecht et al. 2018) and Chinese (Xu et al. 2017) adaptations of the scales were employed based on the English version (Williams 2009; Williams et al. 2000). Statistical analyses indicated that ERP effects were not influenced by the factor of “language” (see Table S2 for more details).

At the end of the experiment, participants received a comprehensive debriefing and provided their informed consent once again. As compensation for their participation in the entire task—lasting approximately 90 min—participants received credit points or a cash payment (€ 30).

### 2.3 EEG Recording and Preprocessing

EEG data were recorded using eight active Ag/AgCl electrodes (AFz, Fz, F3, F4, Cz, Pz, P7 and P8) placed at the frontal, central, and parietal locations. These electrodes were embedded in an elastic cap (EASYCAP, Herrsching, Germany) and filled with conductive gel (Abralyt 2000, EASYCAP). The ground electrode was positioned at FCz, and electrodes attached to the earlobes served as the reference. The impedance for all EEG electrodes was kept below 10 kΩ. Care was also taken to ensure that differences between electrodes did not exceed 2 kΩ for any individual participant. In addition, vertical and horizontal electrooculogram (EOG) signals were recorded to control for ocular artifacts, with the impedance kept below 30 kΩ. These electrodes were then connected to a digital amplifier (BrainAmps amplifier, BrainProducts, Gilching, Germany; input impedance 10 MΩ). During online recording, the EEG data were band‐pass filtered (0.1–100 Hz with 6 dB/Oct), notch‐filtered (50 Hz), and sampled at 500 Hz.

Offline preprocessing of the EEG data was performed using the “Brain Vision Analyzer” (version 2.1, Brain Products, Gilching, Germany). The EEG data were not re‐referenced. The EEG signals were segmented based on the onset of the participants' ball reception (−100 to 800 ms). Each epoch was then filtered (IIR filter: 0.3–30 Hz, 24 dB/Oct) and baseline‐corrected (−100 to 0 ms).

Artifact detection and rejection followed a two‐step procedure—also applied in previous studies (Fang et al. 2022): In the first step, automatic artifact detection was applied, relying on the parameters defined in the analysis software: Trials with ocular artifacts were marked (EOGs > 50 μV), as well as trials with a high deflection in the EEG channels (EEG > 80 μV). Furthermore, trials were marked in which a lack of variance in the EEG activity for at least 200 ms was marked as an override. In the second step, a manual control for artifacts was applied: Here, trials were marked with prominent EEG alpha activity (8–12 Hz) that affected the baseline, and that was larger than the EEG activity in the time windows of interest (200–600 ms). Moreover, linear drifts were identified that exceeded 40 μV and extended over 200 ms. Finally, we checked for high‐frequency bursts.

As in the previous studies, marked trials were excluded from analysis. A rigorous artifact rejection process is essential because shifts in baseline, such as alpha fluctuations, can significantly impact the expression of ERP components. Notably, these effects were more prevalent toward the end of each block.

Consequently, 47.97% of the trials were discarded in block 1 (*n* = 66), resulting in a mean number of artifact‐free trials of 34.34 (CG: *M* = 34.04, range 18–55 trials; EG1_disc_: *M* = 32.87, range 15–56 trials; EG2_cont_: *M* = 35.73, range 19–61 trials). In block 2 (*n* = 92), the mean number of trials included in the analysis was 48.21 (CG: *M* = 45.39, range 20–80 trials; EG1_disc_: *M* = 48.04, range 25–83 trials; EG2_cont_: *M* = 50.52, range 23–89 trials), with a similar rejection rate of 47.76%. The significant increase in artifact‐free trials in block 2 as compared to block 1 (*t*(68) = −8.547, *p* < 0.001, *d* = −1.03, *BF*_−0_ > 100) was due to the experimental manipulation: With the transition to overinclusion, the number of relevant events shifted—in each group—from 66 to 92.

### 2.4 Statistical Analysis

All analyses were conducted using SPSS (version 27, IBM) and Jamovi (version 0.9.4.2, Jamovi Development Team).

#### 2.4.1 Self‐Reported Data

In the first step of the analysis, a manipulation check was performed to assess whether participants noticed the changes in the frequency of their ball reception between block 1 and block 2. This involved a 3 × 2 analysis of variance (ANOVA) with the between‐factor “group” (CG, EG1_disc_ and EG2_cont_) and the within‐factor “block” (block 1 and block 2). The ANOVA results were reported with Greenhouse–Geisser corrected degrees of freedom and *p*‐values.

To test our experimental hypotheses, in the second step of the analysis, each scale of interest (“belonging”, “control”, “negative mood”, “personal power” and “social power”) was analyzed separately using one‐way ANOVAs with the between factor “group” (CG, EG1_disc_ and EG2_cont_). A larger mean difference score (Table 1) indicates greater changes in feelings between the two successive blocks. Reported degrees of freedom and *p*‐values were adjusted using the Greenhouse–Geisser correction. Significant effects from the ANOVAs were further examined through Bonferroni‐corrected post hoc analyses.

**TABLE 1:** Descriptive statistics and ANOVA results for self‐reports and ERP responses in three groups. The first few rows refer to the estimated frequencies of participants' ball receptions and experiences of loss of control. The data for self‐reported threats to “control” and “belonging”, “negative mood”, “personal power” and “social power” are presented as differential scores [Δ (block 2 − block 1)], with positive values indicating greater expression in block 2 and negative values indicating greater expression in block 1. For the ERP components, the values given are the mean voltages of frontal P2 or centro‐parietal P3 within specific time ranges in each block. The last few columns show the results of the ANOVAs: the main effect of the repeated measures ANOVA with the factor “block” on the estimated frequency of ball reception, the effects of the one‐way ANOVAs on the corresponding scales, and the interaction effects for the ERPs in the repeated measures ANOVA with the factors “group” and “block”. Please note that the supplement (Section 4) provides a corresponding table including the confidence intervals.

	CG (*n* = 23)	EG1disc (*n* = 23)	EG2_cont_ (*n* = 23)	Group differences
	*M (SE)*	*M (SE)*	*M (SE)*	*F*
**Estimated frequency (%)**				
*BR*				
B1	33.91 (2.38)	31.35 (2.38)	32.74 (2.38)	49.48***
B2	52.65 (3.59)	49.22 (3.59)	45.48 (3.59)	
*LoC*				
B1	21.17 (4.19)	31.22 (4.19)	22.13 (4.19)	N/A
B2	33.13 (5.09)	11.74 (5.09)	40.26 (5.09)	
NTQ: belonging	0.14 (0.17)	0.70 (0.14)	0.06 (0.19)	4.31*
NTQ: control	0.64 (0.33)	1.23 (0.20)	0.36 (0.23)	2.95
Negative mood	0.07 (0.47)	−2.04 (0.52)	0.09 (0.49)	6.13**
Personal power	0.35 (0.26)	0.13 (0.19)	0.33 (0.21)	0.28
Social power	0.50 (0.32)	1.04 (0.23)	0.67 (0.25)	1.08
**Mean values (μV)**				
*P2*				
B1	0.53 (0.46)	2.29 (0.46)	2.45 (0.46)	16.29***
B2	2.14 (0.42)	1.14 (0.42)	2.27 (0.42)	
*P3*				
B1	4.40 (0.61)	4.77 (0.61)	4.37 (0.61)	1.11
B2	3.39 (0.37)	2.86 (0.37)	3.13 (0.37)	

*Note:* significance levels are coded: *p < 0.05; **p < 0.01; ***p < 0.001.

Abbreviations: B1, block 1; B2, block 2; BR, ball reception; CG, control group without preexposure; CI, confidence interval (95%); EG1disc, experimental group 1 with discontinued first threat; EG2_cont_, experimental group 2 with continued threat; LoC, loss of control; M, mean; N/A, not applicable; NTQ, the need threat questionnaire; SE, standard error.

#### 2.4.2 ERPs Data

The analysis of ERP results focused on the ERPs triggered by the event “ball reception of the participant” which probed the subjective expectancy status. In line with the grand‐averaged ERPs shown in Figure 3A, ERPs were separately analyzed considering the between‐factor “preexposure status” (CG, EG1_disc_ and EG2_cont_) and the within‐factors “block” (block 1 and block 2). The choice of the electrode clusters was defined a priori: The analysis of the P3 effect was focused on a centro‐parietal cluster comprising the electrodes Cz, Pz, P7, and P8. The same cluster definition was used in the aforementioned exclusion study (Fang et al. 2022), and enabled us to provide a comparison of the expression of the P3 effect. Moreover, previous studies have indicated that the centro‐parietal electrode cluster includes the sites most sensitive to a transition‐to‐overinclusion identified in previous overinclusion studies (Niedeggen et al. 2014; Weinbrecht et al. 2018). The analysis of the P2 effect was focused on a centro‐parietal cluster comprising the electrodes AFz, Fz, F3, and F4. Here, the choice of the cluster is based on the reliable expression of the P2 identified in previous studies (Niedeggen et al. 2014; Weinbrecht et al. 2021). Please note, that the definition of these electrode clusters was confirmed a posteriori: The data from the control group (CG, see Figure 3A) confirmed the frontal expression of the P2 effect and the posterior expression of the P3 effect.

The difference amplitude of the grand‐averaged ERP signal (Figure 3B) were chosen to extract the temporal characteristics of the ERP effects induced by the transition from block 1 to block 2. As mentioned before, previous ERP studies (Niedeggen et al. 2023; Weinbrecht et al. 2018, 2021) identified the P3 effect at centro‐parietal leads in the time range of 290 to 420 ms and the P2 effect at fronto‐central leads in the time range from 160 to 250 ms. Based on these templates and the global field power (see Figure S1), a frontal positive wave (P2) peaking at about 230 ms and a centro‐parietal positive wave (P3) that peaks at about 390 ms were identified in the current data.

Taking into account interindividual differences, the time windows defining the mean amplitude for these components were defined as follows: P2 (200–260 ms) and P3 (360–420 ms). Both components were analyzed sequentially by performing 3 × 2 ANOVAs including the factors “Group” (CG, EG1_disc_ and EG2_cont_) and “Block” (block: block 1 and block 2) in the corresponding electrode clusters to test our hypotheses.

In addition, three further sets of statistical analyses are provided in the Data S1 (see Section 6):

First, the a posteriori analysis of the grand‐averaged ERP revealed that the P3 effect appeared to be more sustained in the experimental groups compared to the control group. To account for this difference, we ran an additional analysis of the P3 effect using a prolonged time window (350–450 ms).

Second, in order to check the reliability of the mean amplitude data results, the results for the P2 and P3 were additionally analyzed by running peak analyses. Using the time windows defined above, we performed automatic peak detections (Brain Vision Analyzer) in each experimental block. For the single participants, the positive maxima can be identified in the time range of 360–420 ms (P3, centro‐parietal) and 200–260 ms (P2, anterior‐frontal). The results of the peak detection were further manually checked and verified. The baseline‐related amplitudes with related latency of the P3 and P2 peaks, respectively, were included in a statistical analysis. For each peak, the amplitude and latency were individually analyzed by running a 3 (group: CG, EG1_disc_ and EG2_cont_) × 2 (block: block 1 and block 2) ANOVA. Results are reported with Greenhouse–Geisser corrected degrees of freedom and *p*‐values. Post hoc comparisons were motivated by significant interactions of the experimental factors.

Third, the grand‐averaged data also indicated a posteriorly transient negative‐going wave (N2) that peaks at about 190 ms. This N2 component plays a critical role in attention and monitoring conflict, as reported in earlier Cyberball‐EEG studies (Fang et al. 2022; Mills et al. 2023; Niedeggen et al. 2023). The N2 peak and latency were also analyzed running 3 (group: CG, EG1_disc_ and EG2_cont_) × 2 (block: block 1 and block 2) ANOVAs. Significant interactions between experimental factors were followed by post hoc comparisons.

Please note that we additionally ran *Bayesian* statistics for the self‐report and ERP data. In this case, the corresponding BF_10_ values are provided—comparing to the null model. All tests were performed using JASP 0.19 (JASP team, 2024).

## 3 Results

### 3.1 Manipulation Check

Participants, independent of group assignment, noticed that they received the ball more frequently in the overinclusion block (block 2) compared to the inclusion block (block 1) (see Table 1). An ANOVA revealed a significant main effect of “block”, *F*(1, 66) = 49.48, *p* < 0.001, *η*_p_^2^ = 0.428, *BF*_10_ > 100. The analysis did not show a significant main effect of “group”, *F*(2, 66) = 0.90, *p* = 0.412, *η*_p_^2^ = 0.027, *BF*_10_ = 0.150, or a significant interaction between “block” and “group”, *F*(2, 66) = 0.64, *p* = 0.530, *η*_p_^2^ = 0.019, *BF*_10_ = 0.204. The results confirmed that participants reliably recognized the increase of ball reception in the overinclusion block (block 2) compared to the inclusion block (block 1).

### 3.2 Self‐Reports

As for the self‐reports, we hypothesized that effects on the scales of “belonging” and “negative moods” are primarily related to the impact of the offset of the preexposure threat.

**FIGURE 2:**
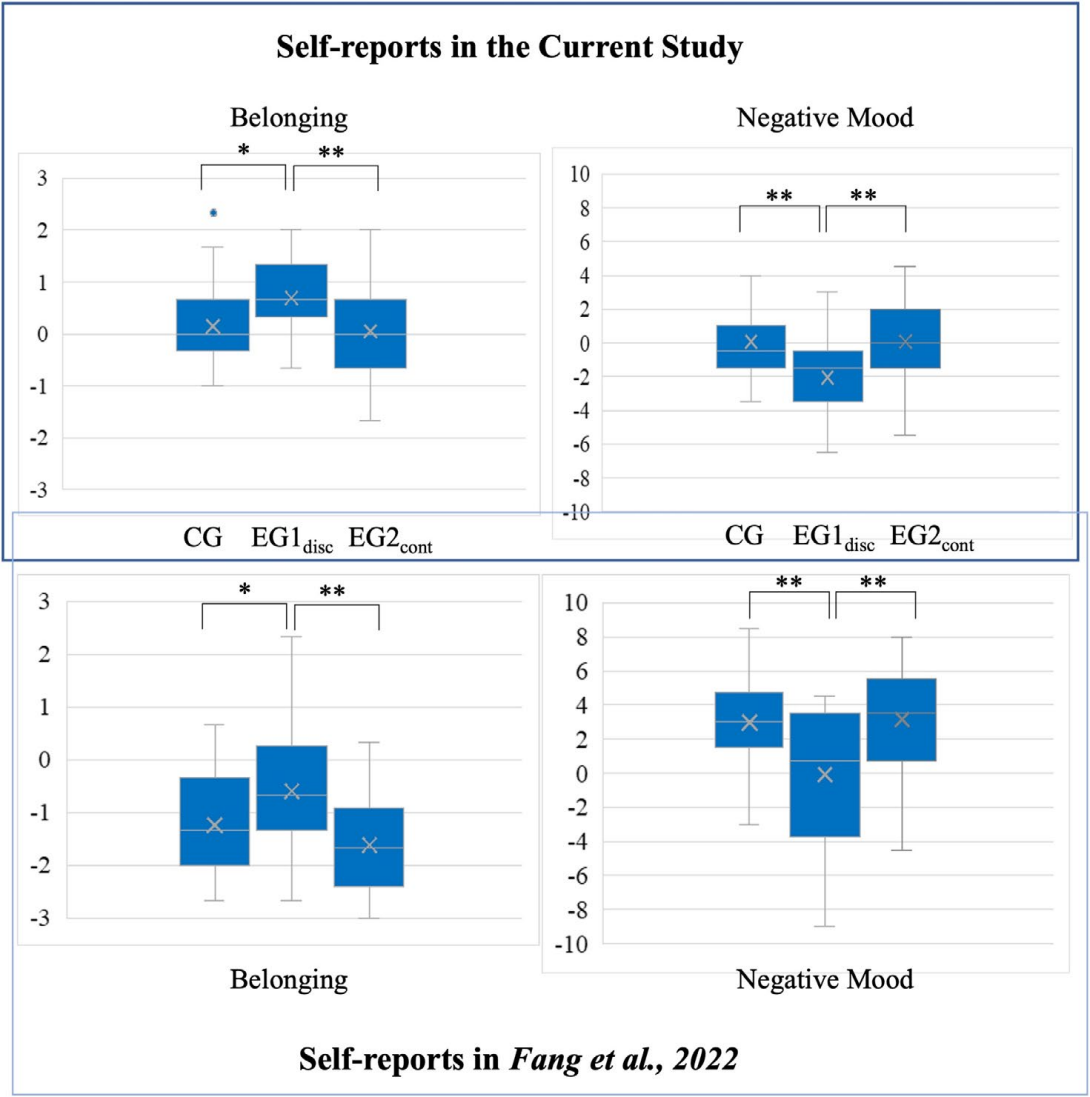
Effects on self‐reported scales. Difference score [Δ (block2 − block1)] in retrospective self‐reports in all groups. The current self‐reports were compared those with of a previous study (Fang et al. 2022). Box‐and‐whisker plots were used in all graphs. The asterisks indicate statistically significant differences between experimental groups. CG, Control group without preexposure; EG1_disc_, Experimental group 1 with discontinued first threat; EG2_cont_, Experimental group 2 with continued first threat. *p < 0.05; **p < 0.01.

As Figure 2 (upper panel) illustrates, a corresponding pattern can be observed in our data: Participants in EG1_disc_ perceived less threat to the need for “belonging” as compared to those in the CG and EG2_cont_. The one‐way ANOVA indicated a significant difference between the three groups, *F*(2, 66) = 4.31, *p* = 0.017, *η*_p_^2^ = 0.116, *BF*_10_ = 3.029. The subsequent post hoc comparisons confirmed that participants in EG1_disc_ reported the highest score on the need to “belonging” during the transition to overinclusion, and that the effects were not significantly different between CG and EG2_cont_ (EG1_disc_ vs. CG, *F*(1, 44) = 6.41, *p* = 0.015, *η*_p_^2^ = 0.127, *BF*_10_ = 3.577, EG1_disc_ vs. EG2_cont_, *F*(1, 44) = 7.32, *p* = 0.010, *η*_p_^2^ = 0.143, *BF*_10_ = 5.011, CG vs. EG2_cont_, *F*(1, 44) = 0.12, *p* = 0.732, *η*_p_^2^ = 0.003, *BF*_10_ = 0.307.) The *Bayesian* statistics indicated substantial evidence of the effects observed in the self‐reports.

A similar pattern was observed for the scale “negative mood”: The significant main effect of the factor “group”, (*F*(2, 66) = 6.13, *p* = 0.004, *η*_p_^2^ = 0.157, *BF*_10_ = 11.234), was due to a substantial change in mood (negative to positive) which was exclusively expressed in the EG1_disc_, but not in the other groups. This is supported by the post hoc test (EG1_disc_ vs. CG, *F*(1, 44) = 9.12, *p* = 0.004, *η*_p_^2^ = 0.172, *BF*_10_ = 9.701, EG1_disc_ vs. EG2_cont_, *F*(1, 44) = 8.86, *p* = 0.005, *η*_p_^2^ = 0.168, *BF*_10_ = 8.811, CG vs. EG2_cont_, *F*(1, 44) = 0.001, *p* = 0.975, *η*_p_^2^ < 0.001, *BF*_10_ = 0.292). The *Bayesian* statistics indicated substantial evidence of the effects observed in the self‐reports.

Regarding other related scales (see Table 1), the one‐way ANOVA did not reveal any significant differences among the three groups for variables such as the need threat to “control”, *F*(2, 66) = 2.95, *p* = 0.059, *η*_p_^2^ = 0.082, *BF*_10_ = 1.112, “personal power”, *F*(2, 66) = 0.28, *p* = 0.754, *η*_p_^2^ = 0.009, *BF*_10_ = 0.151, or “social power”, *F*(2, 66) = 1.08, *p* = 0.347, *η*_p_^2^ = 0.032, *BF*_10_ = 0.274.

### 3.3 ERP Effects: Mean Amplitudes

**FIGURE 3:**
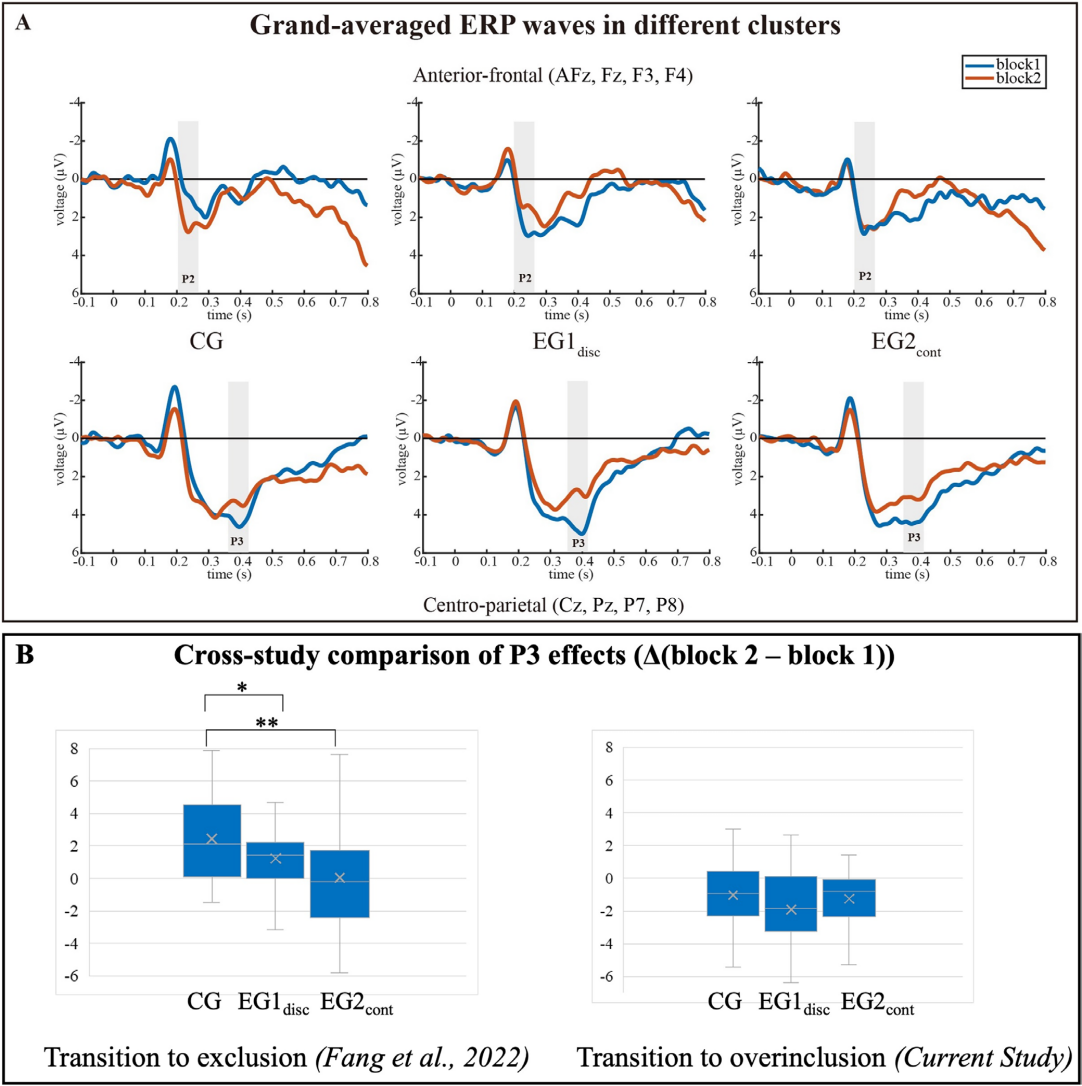
(A) ERP effects at different electrode leads (electrodes clustered). Upper vs. lower panel: The upper panel contrasts the ERPs elicited by the anterior‐frontal electrode leads in block 1 and block 2. The lower panel presents the ERPs elicited by the centro‐parietal electrode leads in block 1 and block 2. Left vs. central vs. right panel: The ERP effects for both clusters are depicted separately for the three experimental groups (CG: Left panel; EG1_disc_: Central panel; EG2_cont_: Right panel). The time windows considered for the analysis of the P2, P3, and N2 effects are highlighted. (B) Differential P3 amplitudes (Δ(block 2 − block 1)) were presented for three groups in the current study and a previous study (left panel: 360–420 ms in the current study; right panel: 400–500 ms in the previous study (Fang et al. 2022)). CG, Control group without preexposure; EG1_disc_, Experimental group 1 with discontinued first threat; EG2_cont_, Experimental group 2 with continued first threat.

The grand‐averaged ERP responses separated for the three groups are presented in Table 1 and Figure 3A. These responses exhibit distinct temporal patterns. A transient negativity is immediately followed by a transient positivity (P2) peaking at about 230 ms and spanning from 200 to 260 ms post‐stimulus. Subsequently, a second positive deflection (P3) peaking at about 290 ms is observed consistently from 360 to 420 ms post‐stimulus (see “2. Methods” for details).

As for the P3 effect (Δ (block 2 − block 1)), a decrease in amplitude with increasing ball reception probability was observed across the three groups (see Figure 3A). Compared to CG, the P3 effect was mostly expressed in EG1_disc_, while only a slight increase was observed in EG2_cont_ (see Figure 3B). A significant main effect of “block” was detected, *F*(1, 66) = 29.73, *p* < 0.001, *η*_p_^2^ = 0.311, *BF*_10_ > 100, indicating that the amplitudes in block 1 were greater than in block 2 across all three groups. However, this effect was not significantly modulated by the “group” assignment, *F*(2, 66) = 1.11, *p* = 0.334, *η*_p_^2^ = 0.033, *BF*_10_ = 0.282. Please note that these effects were confirmed in the analysis of the P3 effect based on a prolonged time window (350–450 ms) as well as for the peak analysis (See details in Data S1).

The P2 was mostly expressed at the frontal leads. Previous experiments indicated that its amplitude was significantly increased by a transition to overinclusion (Niedeggen et al. 2014; Weinbrecht et al. 2021) and labeled this as the “P2 effect”. In our current study, the P2 effect (Δ (block 2 − block 1)) was replicated for the CG with a transition to overinclusion, but decreased in EG1_disc_ and remained relatively unchanged in EG2_cont_ (see Figure 3A).

This descriptive pattern was confirmed by the statistical analysis. Most importantly, the ANOVA did not reveal a significant main effect of the factor “block”, *F*(1, 66) = 0.22, *p* = 0.641, *η*_p_^2^ = 0.003, *BF*_10_ = 0.192, but the crucial significant interaction between the factors “group” and “block”, *F*(2, 66) = 16.29, *p* < 0.001, *η*_p_^2^ = 0.330, *BF*_10_ > 100. In the post hoc test, we focused on the question of whether the expression of the P2 effect (block 2 − block 1) was expressed differently—as indicated by the grand‐averaged data. The statistical test confirmed a gradual decrease in the P2 effect, which was significantly stronger in the CG as compared to both experimental groups, and reversed in the EG1_disc_ (CG vs. EG1_disc_, *F*(1, 44) = 25.30, *p* < 0.001, *η*_p_^2^ = 0.365, *BF*_10_ > 100, CG vs. EG2_cont_, *F*(1, 44) = 15.50, *p* < 0.001, *η*_p_^2^ = 0.261, *BF*_10_ = 88.532, EG2_cont_ vs. EG1_disc_, *F*(1, 44) = 4.39, *p* = 0.042, *η*_p_^2^ = 0.091, *BF*_10_ = 1.662). The *Bayesian* statistics indicated decisive evidence of the effects observed in the P2 effect.

## 4 Discussion

This study investigated the effects of preexposure to loss of control on responses to overinclusion in the Cyberball game. In line with prior findings (Niedeggen et al. 2014; Weinbrecht et al. 2018), the transition from inclusion to overinclusion significantly decreased centro‐parietal P3 amplitude (P3 effect). However, contrary to *Hypothesis 1*, this P3 effect was not significantly influenced by group allocation. Therefore, the current data do not support the idea that ERP correlates of expectancy violation elicited by overinclusion are modulated by preexposure. A modulation of ERP components by preexposure was only expressed for the frontal positivity: Only in the CG, the expected increase in amplitude induced by overinclusion was found (P2 effect), but not in the preexposure conditions (EG1_disc_ and EG2_cont_). Moreover, *Hypothesis 2* was supported: Reduced threat to belongingness and negative mood were only reported by participants for whom the onset of overinclusion was associated with the offsetting of a social threat (EG1_disc_). Conversely, the mere onset of overinclusion (CG) did not enhance the self‐reported feeling of belonging or negative mood. The following sections provide an in‐depth discussion of these findings, incorporating both ERP data and subjective measures.

### 4.1 ERPs Effects

The results confirmed previous findings showing that a transition to overinclusion can lead to decreased P3 amplitudes (Niedeggen et al. 2014; Weinbrecht et al. 2018). The decrease—as contrasted to an increase of the P3 effect elicited by transition to exclusion—appeared to be in line with the assumption of the sociometer theory (Leary et al. 1995), which defines exclusion and overinclusion as endpoints on a scale. However, the modulatory effect of preexposure can only be observed for a transition to exclusion (Fang et al. 2022), but not for a transition to overinclusion (see Figure 3B). That is, the expectancy bias induced by preexposure is not observed if the upcoming social interaction is more positive (here, overinclusion).

The lack of a modulation of the P3 effect can probably be attributed to two accounts. The first one is related to differences in the sensitivity of the ERP effect: The P3 effect is probably more sensitive for shifts towards exclusion as compared to shifts towards overinclusion. This idea is in line with previously reported asymmetry in the response of the P3 amplitude to the target frequency: The P3 amplitude responds more sensitively to a reduced target probability (amplitude increase) as compared to an increase in target probability (amplitude decrease) (Polich et al. 1991; Verleger 2020). A recent meta‐analytic review (Hay et al. 2023) also posited that the effect sizes of overinclusion for self‐reports were smaller than the corresponding effects of exclusion. Higher sensitivity to exclusion compared to overinclusion may be rooted in evolutionary survival mechanisms (Baumeister and Leary 1995; MacDonald and Leary 2005), whereby social connections were crucial for safety and resource acquisition, thus making the pain of exclusion a significant adaptive response. Overall, these findings suggest that the idea of a continuum ranging from exclusion to overinclusion (Leary and Baumeister 2000; Niedeggen et al. 2014; Williams et al. 2000) requires critical reflection.

Related to this account, a second explanation implies that different cognitive processes are signaled by the modulation of the P3 effect in the case of exclusion and overinclusion: In the case of a transition to exclusion, we assume that the readjustment of the a priori expectation on involvement is triggered (Niedeggen et al. 2023; Schuck et al. 2018; Weschke and Niedeggen 2015). This process is more expressed if the expectation is consolidated by a preceding inclusionary phase: An immediate onset of exclusion—not preceded by inclusion—reduces the P3 effect (effect of order: Gutz et al. 2011). Notably, this “order effect” was not obtained in a corresponding overinclusion study (Niedeggen et al. 2014): The P3 effect was not modulated by the order of conditions (inclusion‐to‐overinclusion vs. overinclusion‐to‐inclusion). In other words, a transition to overinclusion doesn't seem to require a readjustment of the existing expectancy of involvement and is probably a mere reflection of changes in target probability.

The latter idea is in line with the findings that overinclusion is associated with another cognitive process—as reflected in the frontal P2 effect (Niedeggen et al. 2014; Weinbrecht et al. 2021). The increase in amplitude of this component has not been related to the processing of social participation: please note that a P2 effect has not been reliably observed in previous exclusionary studies (Vanhollebeke et al. 2023). This component has been rather related to the processing of social rewards (Cao et al. 2015; Distefano et al. 2018; Holroyd et al. 2011; Potts et al. 2006). Supporting this idea, functional magnetic resonance imaging (fMRI) studies have shown activation of the ventral striatum, a region closely related to pleasure enhancement and social reward processing, with increased frequency of social interaction in the Cyberball task (Kawamichi et al. 2016). More importantly, the P2 effect was significantly modulated by the preexposure: In contrast to the control group, the effect was reduced in both experimental groups. Figure 3A suggests that this modulation effect results from the already enhanced P2 amplitudes in the experimental groups in the preexposure condition (block 1). The experienced loss of control in block 1 might facilitate an avoidance response (Young 1959), which reduces the participants' reward sensitivity for the overinclusion in block 2 (Brown et al. 2021; Kohls et al. 2013). Notably, the P2 effect was also differently expressed between the experimental groups: In case of the discontinuation of the preexposure threat, the P2 effect was significantly decreased. We tentatively suggest that this reduction is related to the positive impact of the offset of the aversive preexposure threat (here: intervention) which might overshadow the processing of the overinclusion in block 2. This idea is supported by the self‐reports which also indicate that negative mood is exclusively decreased by the offset of the preexposure threat (see Figure 2).

Although the details of the processes related to the P2 and P3 effects remain to be explored in further studies, our data indicate that preexposure to a social threat does not affect the same cognitive processes in cases of upcoming exclusion or overinclusion. This evidence challenges the notion of a linear continuum of social interaction effects. Instead, these results point in the direction of the existence of two distinct value systems: As proposed by Yacubian et al. (2006), the ventral striatum is exclusively involved in processing the gain‐related aspects of expected value, whereas the amygdala is responsible for processing loss‐related expected value and the associated prediction error. In other words, overinclusion might link to positive expectations (associated with the ventral striatum), while social threats might tie to negative expectations (associated with the amygdala).

### 4.2 Self‐Reported Effects

Previous studies on overinclusion have shown mixed self‐reported results: some found that overinclusion decreases the threat to belonging and negative mood beyond fair inclusion (De Waal‐Andrews and Van Beest 2020; Niedeggen et al. 2014; Venturini et al. 2016), while others did not report significant differences (Kawamoto et al. 2012; van Beest and Williams 2006). The results of the control group in this study support the latter: As Figure 2 shows, the transition to overinclusion did not result in a significant decrease in need threat to belonging or negative mood.

Despite the lack of an effect in the control group, our data revealed that the preexposure could affect the expression of the self‐reports. Most importantly, we see a response pattern that is congruent with previous results: Figure 2 allows a comparison of the expression of the experienced threat for “belonging” and “negative mood” observed in the current and a previous preexposure study (Fang et al. 2022). In both studies, the offset of the preexposure threat leads to a decreased threat to belonging and negative mood during the transition to exclusion or overinclusion. The distribution of means after *z*‐score transformation in both studies (see Figure S2) confirmed this impression. This finding confirms our second hypothesis: the offset of the preexposure threat in the current study, as well as in a previous study (Fang et al. 2022), seems to reflect a regaining of control, thereby improving individuals' affective state and, consequently, affecting the need for belonging. This idea also aligns with the self‐determination theory (Deci and Ryan 2000), suggesting that perceived control over one's actions and decisions can foster positive outcomes across various life domains.

## 5 Limitations

Several important limitations must be considered when interpreting these findings:

First, the results of this study suggest that individuals are more sensitive to exclusion than to overinclusion. However, the frequencies of the overinclusion scenarios (46%, 13% higher than the equity condition) in this study and the exclusion scenarios (17%, 16% lower than the equity condition) in the previous study (Fang et al. 2022) do not exactly match, which may have contributed to the observed difference. Future studies should ensure that these frequencies are fully matched to accurately assess sensitivity levels.

Second, to be able to compare this study with previous research (Fang et al. 2022), we also applied loss of control as a preexposure stimulus and introduced a “supervisor” character. Although participants in the control group did not experience any loss of control due to interventions of the supervisor, participants still reported perceiving loss of control (see Table 1). This may be due to participants' potential uncertainty about whether they had experienced interference, which may have led to inaccurate self‐reports. Future studies might consider setting up several intervention events during the practice phase so that participants know exactly what the interfered ball throw looks like.

Third, the relative judgment in self‐reports does not account for baseline differences between the groups. That is, it is likely that some feelings were already influenced by the preexposure threat in block 1. By using the first block as a baseline, the focus of comparison may have shifted solely to improvements in participants' social inclusion status in the continuous presence (EG2_cont_) or absence (CG) of preexposure threats, whereas participants in EG1_disc_ perceived a broader range of positive emotional changes (i.e., improvement in participants' social inclusion status *and* regaining of control). To address this limitation, future research could include an additional baseline block before the experimental blocks, allowing for a more accurate assessment and comparison of self‐reported responses across groups.

Finally, the homogeneity of our participant pool, consisting predominantly of undergraduate students from different native‐speaking countries, with similar educational backgrounds and specific demographic profiles, may have influenced their responses to social interaction. Future research with a more diverse and representative sample is needed to increase the external validity and applicability of our findings.

## 6 Conclusions

The current study indicates that the effect of preexposure threats observed in an exclusionary Cyberball (Fang et al. 2022) cannot be transferred to an overinclusion setting: The ERP effects related to expectancy violation (Weinbrecht et al. 2018; Weschke and Niedeggen 2015) are not triggered, while ERP effects related to social reward processing (Cao et al. 2015; Kawamichi et al. 2016; Niedeggen et al. 2014; Weinbrecht et al. 2021) are enhanced—and modulated by the experimental conditions. The results therefore question the idea of an internal continuum in the processing of social participation. They also imply that the experience of overinclusion has an ameliorative effect on the consequences of ostracism (DeWall and Bushman 2011; Williams and Nida 2011).

## Author Contributions

**Xu Fang:** conceptualization, data curation, formal analysis, software, visualization, writing – original draft. **Rudolf Kerschreiter:** conceptualization, funding acquisition, project administration, writing – review and editing. **Yu‐Fang Yang:** data curation, software, writing – review and editing. **Michael Niedeggen:** conceptualization, data curation, formal analysis, funding acquisition, methodology, project administration, software, writing – review and editing.

## Ethics Statement

The research protocol was approved by the local ethics committee of Freie Universität Berlin (No. 006.2019, May 15, 2019).

## Consent

Before and after the experiment, all participants gave written informed consent according to the Declaration of Helsinki.

## Conflicts of Interest

The authors declare no conflicts of interest.

## Supporting information


Data S1.

## Data Availability

Preprocessed EEG data, self‐reported data, questionnaires, as well as the source code of the experimental procedure are available here: https://osf.io/qpajf/.
