# Supplementary material for: Electrophysiological and Behavioral Responses to Overinclusion Following Preexposure to Social Threat
Source: Psychophysiology. 2025 Jul 5;62(7):e70107. doi: 10.1111/psyp.70107 (PMC12228052; doi:10.1111/psyp.70107)
Supplement: Supplementary file 1 — Data S1. [file PSYP-62-e70107-s001.docx]

***Supplementary Material***

**Electrophysiological and behavioral responses to overinclusion following preexposure to social threat**

Xu Fang^1,2^, Rudolf Kerschreiter^3^, Yu-Fang Yang^2^, Michael Niedeggen^2*^

^1^Institute of Psychology and Behavior, Henan University, Kaifeng, China

^2^Division of Experimental Psychology and Neuropsychology, Department of Education and Psychology, Freie Universität Berlin, Berlin, Germany

^3^Division of Social, Organizational, and Economic Psychology, Department of Education and Psychology, Freie Universität Berlin, Berlin, Germany

*Corresponding author: michael.niedeggen@fu-berlin.de

# Section 1: Distribution of participants

All participants with different mother tongue were randomly assigned and equally distributed to the three group. The distribution in different groups is as follows:

***Table S1.*** Distribution of participants. *Notes*. CG: control group without preexposure; EG1_disc_: experimental group 1 with discontinued first threat; EG2_cont_: experimental group 2 with continued first threat.

|  | German | English | Chinese | total |
| --- | --- | --- | --- | --- |
| CG | 3 | 3 | 17 | 23 |
| EG1_disc_ | 4 | 2 | 17 | 23 |
| EG2_cont_ | 2 | 2 | 19 | 23 |
| Total | 9 | 7 | 53 | 69 |

# Section 2: The factor of “language” did not influence the significance of the ERPs effects

***Table S2.*** ***ANOVA results for self-reports and ERP responses in three groups.***

|  | | | **ANOVA** | | | |
| --- | --- | --- | --- | --- | --- | --- |
|  |  |  | ***No covariates*** | | ***language as a covariate*** | |
|  |  |  | ***df*** | ***F*** | ***df*** | ***F*** |
| Mean Values (µV) | P2 | B1 | (2, 66) | 16.29*** | (2, 66) | 15.875*** |
|  |  | B2 |  |  |  |  |
|  | P3 | B1 | (2, 66) | .33 | (2, 66) | .42 |
|  |  | B2 |  |  |  |  |

*Notes. CG: control group without preexposure; EG1_disc_: experimental group 1 with discontinued first threat; EG2_cont_: experimental group 2 with continued threat; significance levels are coded: * p < .05; ** p < .01; *** p < .001.*

# Section 3: Local maxima were identified by global field power (GFP)

**

390 ms

***Figure S1***. Average GFP of difference waves (Δ (block 2 – block 1)) among three groups. Two distinct local maxima were identified: frontal local maximum occurred at 230 ms, and the posterior local maximum occurred at 390 ms.

# Section 4: Table S3 including CI and ANOVA details

***Table S3****. Descriptive statistics and ANOVA results for self-reports and ERP* ***responses*** *in three groups. The first few rows refer to the estimated frequencies of participants’ ball receptions and experiences of loss of control. The data for self-reported threats to “control” and “belonging”, “negative mood”, “personal power” and “social power” are presented as differential scores (Δ (block 2-block 1)), with positive values indicating greater expression in block 2 and negative values indicating greater expression in block 1. For the ERP components, the values given are the mean voltages of frontal P2 or centro-parietal P3 within specific time ranges in each block. The last few columns show the results of the ANOVAs: the main effect of the repeated measures ANOVA with the factor “block” on the estimated frequency of ball reception, the effects of the one-way ANOVAs on the corresponding scales, and the interaction effects for the ERPs in the repeated measures ANOVA with the factors “group” and “block”.*

|  | | | | | **CG (*n* = 23)** | **EG1_disc_ (*n* = 23)** | **EG2_cont_ (*n* = 23)** | **ANOVA** | |
| --- | --- | --- | --- | --- | --- | --- | --- | --- | --- |
|  |  |  |  |  | ***M (SE) [CI (95%)]*** | ***M (SE) [CI (95%)]*** | ***M (SE) [CI (95%)]*** | ***df*** | ***F*** |
| Estimated frequency (%) | | BR | | B1 | 33.91 (2.38) [29.17, 38.66] | 31.35 (2.38) [26.60, 36.09] | 32.74 (2.38) [27.99, 37.48] | (1, 66) | 49.48*** |
|  |  |  |  | B2 | 52.65 (3.59) [45.49, 59.81] | 49.22 (3.59) [42.06, 56.38] | 45.48 (3.59) [38.32, 52.64] |  |  |
|  |  | LoC | | B1 | 21.17 (4.19) [12.81, 29.53] | 31.22 (4.19) [22.86, 39.58] | 22.13 (4.19) [13.77, 30.49] | N/A | N/A |
|  |  |  |  | B2 | 33.13 (5.09) [22.96, 43.30] | 11.74 (5.09) [1.58, 21.91] | 40.26 (5.09) [30.09, 50.43] |  |  |
| NTQ: belonging | | | | | .14 ( .17) [-.20, .49] | .70 ( .14) [ .40, .99] | .06 ( .19) [-.33, .45] | (2, 66) | 4.31* |
| NTQ: control | | | | | .64 ( .33) [-.04, 1.32] | 1.23 ( .20) [ .81, 1.65] | .36 ( .23) [-.11, .84] | (2, 66) | 2.95 |
| Negative Mood | | | | | .07 ( .47) [-.91, 1.04] | -2.04 ( .52) [-3.12, -.97] | .09 ( .49) [-.94, 1.12] | (2, 66) | 6.13** |
| Personal Power | | | | | .35 ( .26) [-.19, .89] | .13 ( .19) [-.27, .53] | .33 ( .21) [-.12, .77] | (2, 66) | .28 |
| Social Power | | | | | .50 ( .32) [-.16, 1.16] | 1.04 ( .23) [ .57, 1.51] | .67 ( .25) [ .15, 1.19] | (2, 66) | 1.08 |
| Mean Values (µV) | P2 | | B1 | | .53 ( .46) [-.38, 1.44] | 2.29 ( .46) [1.37, 3.22] | 2.45 ( .46) [1.52, 3.38] | (2, 66) | 16.29*** |
|  |  |  | B2 | | 2.14 ( .42) [1.31, 2.98] | 1.14 ( .42) [ .31, 1.97] | 2.27 ( .42) [1.44, 3.10] |  |  |
|  | P3 | | B1 | | 4.40 ( .61) [3.21, 5.60] | 4.77 ( .61) [3.55, 5.99] | 4.37 ( .61) [3.17, 5.57] | (2, 66) | 1.11 |
|  |  |  | B2 | | 3.39 ( .37) [2.65, 4.13] | 2.86 ( .37) [2.12, 3.60] | 3.13 ( .37) [2.39, 3.87] |  |  |

*Notes. CG: control group without preexposure; EG1_disc_: experimental group 1 with discontinued first threat; EG2_cont_: experimental group 2 with continued threat; BR: ball reception; LoC: loss of control; B1: block 1; B2: block 2; M: mean; SE: standard error; CI: confidence interval (95%); NTQ: the need threat questionnaire; N/A, not applicable; significance levels are coded: * p < .05; ** p < .01; *** p < .001.*

# Section 5: Table S2. Comparable results for non-matched for number of trials vs. matched for number of trials.

As already done in previous studies (Niedeggen et al., 2023; Schuck et al., 2018) – we can match the signal-to-noise ratio between the blocks to further verify the results. To this end, the number of EEG segments in the condition “ball reception” in block 1 was adjusted to the number of segments in the condition “ball reception” in block 2 by random selection. The results in **Table S4** verified no disruptive biases for this discrepancy.

***Table S4****.* Comparable results for non-matched for number of trials vs. matched for number of trials.

|  | ERPs: Ball reception of participant | |
| --- | --- | --- |
|  | P2 effect (200-260ms) | P3 effect (360-420ms) |
| Statistics:  Non-matched for number of trials | Group x Block  *F*(2, 66) = 16.29, *p* < .001, *η*_p_^2^ = .330 | Group x Block  *F*(2, 66) = 1.11, *p* = .334, *η*_p_^2^ = .033 |
| Statistics:  Matched for number of trials | Group x Block  *F*(2, 66) = 5.84, *p =* .005, *η*_p_^2^ = .150 | Group x Block  *F*(2, 66) = 2.19, *p* = .120, *η*_p_^2^ = .062 |

# Section 6: two further sets of statistical analysis

### **Additional analysis for P3 (time window: 350-450 ms)**

We also noticed that the P3 effect appears to be more extended in the experimental as compared to the control groups. To this end, we run an additional analysis of the P3 effect considering an extended time window (350 – 450 ms). This time window still includes the GFP maximum of the P3 effect, but also its sustained character in the experimental groups. However, the ANOVA did not signal a significant interaction of the experimental factors “block” and “group”, *F*(2, 66) = 0.713, *p* = .494, *η*_p_^2^ = .021, *BF*_10_ = .209.

### **ERP Effects: Peaks for P2 and P3**

The analysis of the ERP peaks was conducted to further verify the outcomes above concerning the mean amplitude within the relevant time windows:

For the P3 peak (see **Table S5**), the results were consistent with the earlier analysis of mean amplitude in the corresponding time window. Specifically, the ANOVA on the amplitude revealed a significant main effect of “block”, F(1, 66) = 33.66, p < 0.001, η_p_^2^ = 0.338, but no significant interaction between “block” and “group”, F(2, 66) = 1.63, p = 0.204, η_p_^2^ = 0.047. Furthermore, the analysis of estimated latencies showed no significant effects, neither in the main effect of “block”, F(1, 66) = 1.81, p = 0.183, η_p_^2^ = 0.027, nor in the interaction effect of “group” and “block”, F(2, 66) = 0.65, p = 0.523, η_p_^2^ = 0.019.

For the P2 peak (see **Table S5**), the results of the peak values mostly align with the results of the mean amplitude in the corresponding time range: The significant interaction was detected between “group” and “block”, F(2, 66) = 15.28, p < 0.001, η_p_^2^ = 0.317. Subsequent post-hoc tests replicated the earlier findings, revealing differences between EG1_disc_ and CG, F(1, 44) = 24.30, p < 0.001, η_p_^2^ = 0.355, also EG2_cont_ and CG, F(1, 44) = 16.3, p < 0.001, η_p_^2^ = 0.270. The difference is that although EG2_cont_ also showed different P2 peak values compared to EG1_disc_, there was no statistical significance between them, F(1, 44) = 3.53, p = 0.067, η_p_^2^ = 0.074. It was worth highlighting that there was a different pattern in the estimated latencies: They increased from block1 to block2 in CG, but decreased in the EG1_disc_ and remained unchanged in EG2_cont_. This led to a significant interaction effect between “group” and “block”, F(2, 66) = 3.61, p = 0.033, η_p_^2^ = 0.099. Subsequent post-hoc tests indicated that EG1_disc_ was significantly different from CG, F(1, 44) = 7.10, p = 0.011, η_p_^2^ = 0.139, but EG2_cont_ was not significantly different from CG, F(1, 44) = 0.75, p = 0.393, η_p_^2^ = 0.017. There was also no significant difference between EG1_disc_ and EG2_cont_, F(1, 44) = 2.82, p = 0.100, η_p_^2^ = 0.060.

In summary, the ERP peak results confirmed the analyses of mean amplitudes in the related time ranges, with consistent patterns between these two types of analyses.

### **ERP Effects: N2**

The inspection of the grand-averaged ERPs also triggered a post-hoc analysis of the N2 component. As shown in **Table S5**, the N2 peak amplitudes were also affected by the transition to overinclusion in all three groups. Specifically, the peak amplitude decreased in CG and EG2_cont_, but increased slightly in EG1_disc_. The results showed that the main factor “block” was significant, F(1, 66) = 6.06, p = 0.016, η_p_^2^ = 0.084. A significant interaction between the factors “group” and “block” was observed, F(2, 66) = 3.63, p = 0.032, η_p_^2^ = 0.099. Post-hoc tests revealed that changes in EG1_disc_ were significantly different as compared to CG, F(1, 44) = 6.62, p = 0.014, η_p_^2^ = 0.131, but no significant differences were found between CG and EG2_cont_, F(1, 44) = 1.79, p = 0.188, η_p_^2^ = 0.039, or between EG1_disc_ and EG2_cont_, F(1, 44) = 2.05, p = 0.160, η_p_^2^ = 0.044. Estimated latencies were also not significant in either the main effect of “block”, F(1, 66) = 0.49, p = 0.486, η_p_^2^ = 0.007, or the interaction effect of “group” and “block”, F(2, 66) = 0.93, p = 0.398, η_p_^2^ = 0.028.

***Table S5.*** Descriptive statistics and ANOVA results for ERP responses in three groups. For each component of interest, the values are provided for the mean, maxima as well as latencies corresponding to the maxima within relative time ranges. For the ANOVAs, the interaction effects of the repeated-measures ANOVA with the factor “group” x “block” were present.

|  | | | **CG (*n* = 23)** | **EG1_disc_ (*n* = 23)** | **EG2_cont_ (*n* = 23)** | **ANOVA** | |
| --- | --- | --- | --- | --- | --- | --- | --- |
|  |  |  | ***M (SE) [CI (95%)]*** | ***M (SE) [CI (95%)]*** | ***M (SE) [CI (95%)]*** | ***df*** | ***F*** |
| Peaks: Values (µV) | P3 | B1 | 5.28 (0.65) [3.99, 6.57] | 5.78 (0.65) [4.49, 7.07] | 5.19 (0.65) [3.90, 6.47] | (2, 66) | 1.63 |
|  |  | B2 | 4.23 (0.42) [3.39, 5.06] | 3.55 (0.42) [2.72, 4.39] | 3.74 (0.42) [2.90, 4.57] |  |  |
|  | P2 | B1 | 1.74 (0.51) [0.72, 2.76] | 3.73 (0.51) [2.72, 4.75] | 3.82 (0.51) [2.80, 4.84] | (2, 66) | 15.28*** |
|  |  | B2 | 3.56 (0.47) [2.62, 4.49] | 2.31 (0.47) [1.38, 3.24] | 3.51 (0.47) [2.58, 4.45] |  |  |
|  | N2 | B1 | -3.32 (0.49) [-4.30, -2.34] | -2.36 (0.49) [-3.34, -1.38] | -2.73 (0.49) [-3.71, -1.75] | (2, 66) | 3.63* |
|  |  | B2 | -2.05 (0.41) [-2.88, -1.22] | -2.54 (0.42) [-3.37, -1.71] | -2.19 (0.42) [-3.02, -1.36] |  |  |
| Peaks: Latency (ms) | P3 | B1 | 391.0 (3.78) [383.4, 398.5] | 388.5 (3.78) [381.0, 396.1] | 389.2 (3.78) [381.7, 396.8] | (2, 66) | 0.65 |
|  |  | B2 | 398.6 (3.34) [391.9, 405.3] | 392.8 (3.34) [386.1, 399.4] | 388.9 (3.34) [382.2, 395.5] |  |  |
|  | P2 | B1 | 227.0 (3.38) [220.2, 233.7] | 233.1 (3.38) [226.4, 239.9] | 232.5 (3.38) [225.8, 239.3] | (2, 66) | 3.61* |
|  |  | B2 | 230.7 (2.87) [225.0, 236.4] | 226.9 (2.87) [221.1, 232.6] | 233.2 (2.87) [227.5, 238.9] |  |  |
|  | N2 | B1 | 189.5 (2.50) [184.5, 194.5] | 192.1 (2.50) [187.1, 197.1] | 186.3 (2.50) [181.3, 191.3] | (2, 66) | 0.93 |
|  |  | B2 | 191.1 (2.60) [185.9, 196.3] | 190.3 (2.60) [185.1, 195.5] | 183.4 (2.60) [178.2, 188.6] |  |  |

*Notes*. CG: control group without preexposure; EG1_disc_: experimental group 1 with discontinued preexposure; EG2_cont_: experimental group 2 with continued threat; B1: block 1; B2: block 2; *M*: mean; *SE*: standard error; *CI*: confidence interval (95%); significance levels are coded: * = *p* < .05; ** = *p* < .01; *** = *p* < .001.

# Section 7: Thee z-transformed data in the present study and previous study (Fang et al., 2022).


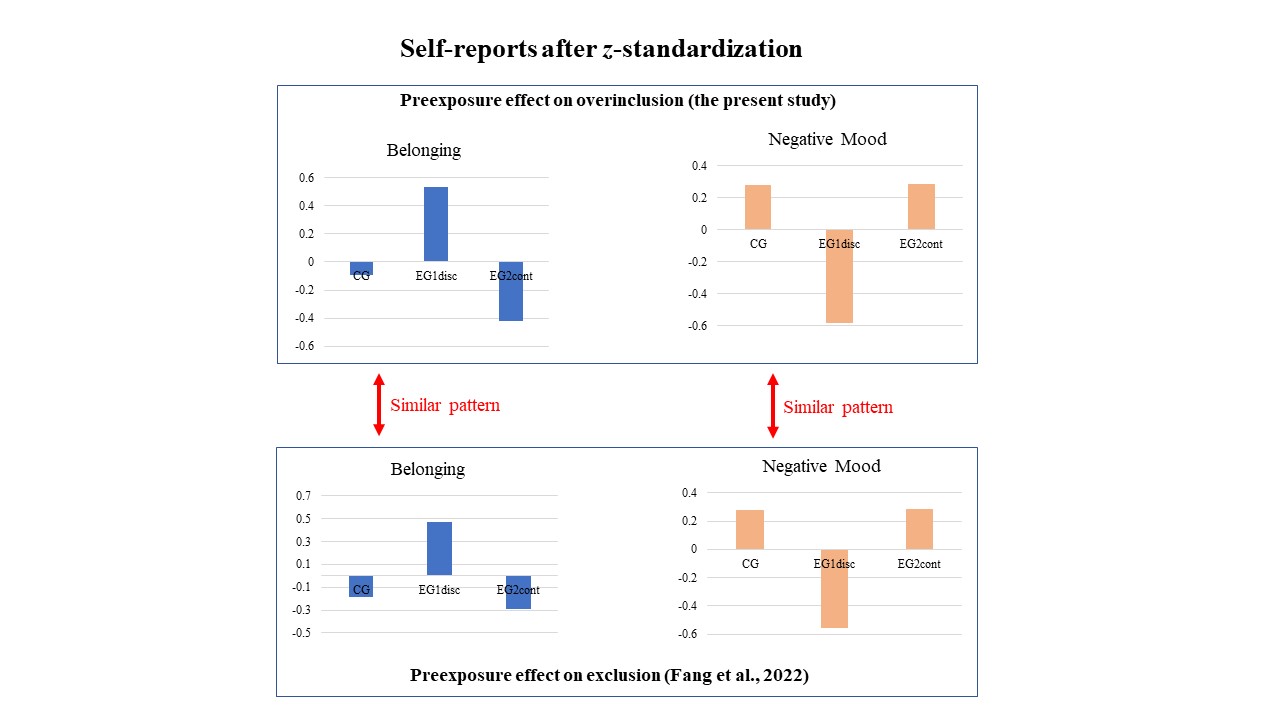


***Figure S2*** Distribution of means after *z*-score transformation. *Notes.* CG: control group without preexposure; EG1_disc_: experimental group 1 with discontinued preexposure; EG2^cont^: experimental group 2 with continued threat;

**References:**

Fang, X., Yang, Y.-F., Kerschreiter, R., & Niedeggen, M. (2022). From Loss of Control to Social Exclusion: ERP Effects of Preexposure to a Social Threat in the Cyberball Paradigm. *Brain Sciences, 12*(9), 1225. <https://doi.org/10.3390/brainsci12091225>

Niedeggen, M., Fang, X., Yang, Y.-F., & Kerschreiter, R. (2023). Electrophysiological evidence for sensitization effects elicited by concurrent social threats. *Scientific Reports, 13*(1), 12285. <https://doi.org/10.1038/s41598-023-39456-0>

Schuck, K., Niedeggen, M., & Kerschreiter, R. (2018). Violated expectations in the cyberball paradigm: Testing the expectancy account of social participation with ERP. *Frontiers in Psychology, 9*, 1762. <https://doi.org/10.3389/fpsyg.2018.01762>
